# Associations between maternal smoking around birth and hepatocellular carcinoma: A bidirectional two-sample Mendelian randomization study in East Asian populations

**DOI:** 10.18332/tid/218848

**Published:** 2026-04-28

**Authors:** Weifu Liu, Wenchang Yu, Kongzhi Zhang, Ming Wang

**Affiliations:** 1 Department of Oncology and Vascular Interventional Therapy, Clinical Oncology School of Fujian Medical University, Fujian Cancer Hospital, Fuzhou, China; 2 Department of Hepatopancreatobiliary Surgical Oncology, Clinical Oncology School of Fujian Medical University, Fujian Cancer Hospital, Fuzhou, China; 3 Fujian Provincial Key Laboratory of Tumor Biotherapy, Fuzhou, China

**Keywords:** East Asian population, maternal smoking around birth, fetal development, hepatocellular carcinoma, Mendelian randomization

## Abstract

**INTRODUCTION:**

Maternal smoking around birth is known to cause a range of adverse fetal outcomes. This study aimed to investigate the potential relationship between maternal smoking around birth and the susceptibility of offspring to hepatocellular carcinoma (HCC) in East Asian populations.

**METHODS:**

Genome-wide association study (GWAS) summary statistics for maternal smoking around birth, which were obtained from the UK Biobank (ukb-e-1787_EAS, data from 2020), and hepatocellular carcinoma, which were obtained from Biobank Japan (bbj-a-158, data from 2019) in East Asians, were obtained from the Mendelian randomization (MR) database platform. Single-nucleotide polymorphisms (SNPs) strongly associated with maternal smoking around birth were selected as instrumental variables (IVs) for two-sample MR analyses. Three complementary MR approaches were applied: inverse-variance weighting (IVW), weighted median estimation, and MR-Egger regression.

**RESULTS:**

A total of 113 SNPs significantly associated with maternal smoking around birth were identified after rigorous selection. Across all MR methods, consistent evidence supported a positive association between maternal smoking around birth and increased HCC risk in offspring (IVW: OR=1.06; 95% CI: 1.05–1.07; weighted median: OR=1.06; 95% CI: 1.05–1.08; MR-Egger: OR=1.05; 95% CI: 1.02–1.08). Conversely, no evidence supported a potential effect of HCC on maternal smoking around birth in reverse MR, reinforcing the directionality of the observed association.

**CONCLUSIONS:**

This study provides genetic evidence supporting a possible link between increased maternal smoking around birth and elevated HCC risk in offspring among East Asians.

## INTRODUCTION

Smoking is an established independent risk factor for several adverse maternal and perinatal outcomes^1^. Cigarette smoking during pregnancy has been linked to miscarriage^2^, preterm birth^3^, and sudden infant death syndrome^4^. For mothers, smoking increases the risk of breast cancer^5^, osteoporosis^6^, and infertility^7^. Recent studies have also suggested that smoking may elevate the risk of hepatocellular carcinoma (HCC)^8^.

HCC accounts for >80% of all primary liver cancers^9^ and is the sixth most common cancer in men and the eleventh in women worldwide^10^. In female HCC patients, circulating levels of insulin-like growth factor-1 and its binding proteins are significantly reduced, whereas estradiol, testosterone, and sex hormone-binding globulin levels are slightly higher^11^. Although maternal estrogen and progesterone levels undergo predictable fluctuations during pregnancy^12^, whether maternal smoking during pregnancy increases the offspring’s risk of developing HCC remains unclear due to ethical and practical constraints on direct observational studies.

Observational research is inherently limited by confounding, lack of randomization, and potential reverse causation^13^. Although randomized controlled trials are the gold standard for observed inference, they are ethically infeasible in this context. Mendelian randomization (MR), which uses genetic variants as instrumental variables (IVs), offers a powerful alternative for observed inference. Because genetic variants are randomly allocated at conception according to Mendel’s laws, MR analyses are less prone to confounding and reverse causation^14^. The growing availability of genome-wide association studies (GWAS) and meta-analyses has further expanded the utility of MR for examining observed relationships^15^. Two-sample MR leverages GWAS summary statistics from separate but comparable samples to estimate the potential effect of an exposure on an outcome. This study aimed to evaluate the potential relationship between maternal smoking around birth and offspring HCC risk in East Asians, using a bidirectional two-sample MR approach.

## METHODS

### Study design

**Figure 1:**
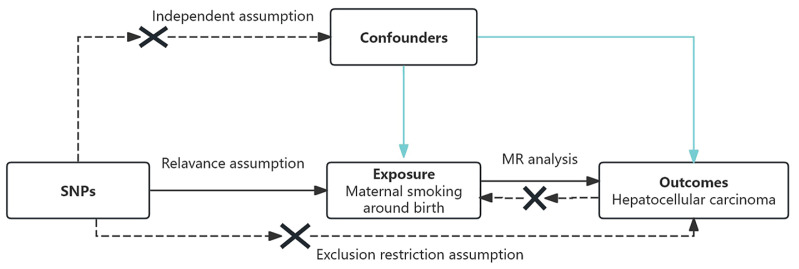
Core design principles of Mendelian randomization

This study followed the core principles and analytical framework of MR (STROBE-MR checklist, Supplementary file)^16^ (Figure 1).

### Instrumental variable selection

To ensure valid MR inference, SNPs were selected according to strict prespecified criteria. First, SNPs were required to reach genome-wide significance for association with the exposure (p<5×10^-8^), although a relaxed threshold (p<10^-6^) was applied when the number of available variants was limited. Second, variants with a minor allele frequency (MAF) >0.01 in the outcome dataset, were retained. Third, linkage disequilibrium (LD) clumping was performed to ensure independence among instruments, using an r^2^ threshold of <0.001 within a 10000 kb window. Fourth, SNPs associated with potential confounders or the outcome were excluded after screening with the *FastTraitR* package, with particular attention to variants associated with viral hepatitis. After completing all filtering steps, 113 independent SNPs were included in the downstream analysis. The proportion of variance explained (R^2^) by each SNP was calculated as R^2^ = [2β^2^×EAF×(1−EAF)]/[2β^2^×EAF×(1−EAF) + 2×SE^2^×N×EAF×(1−EAF)], where β represents the effect size, EAF denotes the effect allele frequency, SE is the standard error, and N is the sample size. Instrument strength was evaluated using the F statistic, calculated as F = [(N−k−1)/k]×[R^2^/(1−R^2^)], where k represents the number of instrumental variables. An F statistic <10 was considered indicative of weak instrument bias, which may result in biased association effect estimates.

### Statistical analysis

Two-sample MR analyses were conducted using R (version 4.3.2) and the *TwoSampleMR* package (version 0.6.14). Three complementary MR approaches were used: IVW^17,18^, weighted median^19^, and MR-Egger regression^20^. Association estimates were reported as odds ratios (ORs) with 95% confidence intervals (CIs). Statistical significance was defined as p<0.05.

### Sensitivity analyses

Robustness of the association estimates was comprehensively evaluated as part of the statistical analysis. Directional horizontal pleiotropy was assessed using the MR-Egger intercept test, with a statistically significant intercept indicating potential pleiotropic effects. Between-instrument heterogeneity was examined using Cochran’s Q statistic. In addition, a leave-one-out analysis was performed to evaluate the influence of individual SNPs on the overall causal estimate by iteratively removing each variant and recalculating the results^21^. Together, these sensitivity analyses ensured the stability and reliability of the MR findings.

## RESULTS

### Data sources

*Exposure*

GWAS summary statistics for maternal smoking around birth were obtained from the UK Biobank (ukb-e-1787_EAS, data from 2020), released in 2020^22^. This dataset included 2406 East Asian samples and 8123409 SNPs.

*Outcome*

GWAS summary statistics for HCC were derived from Biobank Japan (bbj-a-158, data from 2019), based on a large East Asian cohort published in 2020^23^, comprising 197611 samples and 8885115 SNPs. Dataset characteristics are summarized in Table 1.

**Table 1:** Characteristics of the two-sample GWAS datasets, data from 2019 and 2020 (N=2406 for maternal smoking around birth; N=197611 for hepatocellular carcinoma)

*Exposure/outcomes*	*Web source*	*Sample size*	*SNP size*	*Authors*	*Year*	*Population*
Maternal smoking around birth	UK Biobank (ukb-e-1787_EAS)	2406	8123409	Pan-UKB team	2020	East Asian
Hepatocellular carcinoma	Biobank Japan (bbj-a-158)	197611	8885115	Ishigaki et al.^23^	2019	East Asian

GWAS: Genome-Wide Association Study.

### Characteristics of included SNPs

Detailed SNP-level information is presented in Supplementary file Table 1, including effect alleles, allele frequencies, and association estimates for both exposure and outcome datasets.

### Potential effect of maternal smoking around birth on HCC

**Figure 2:**
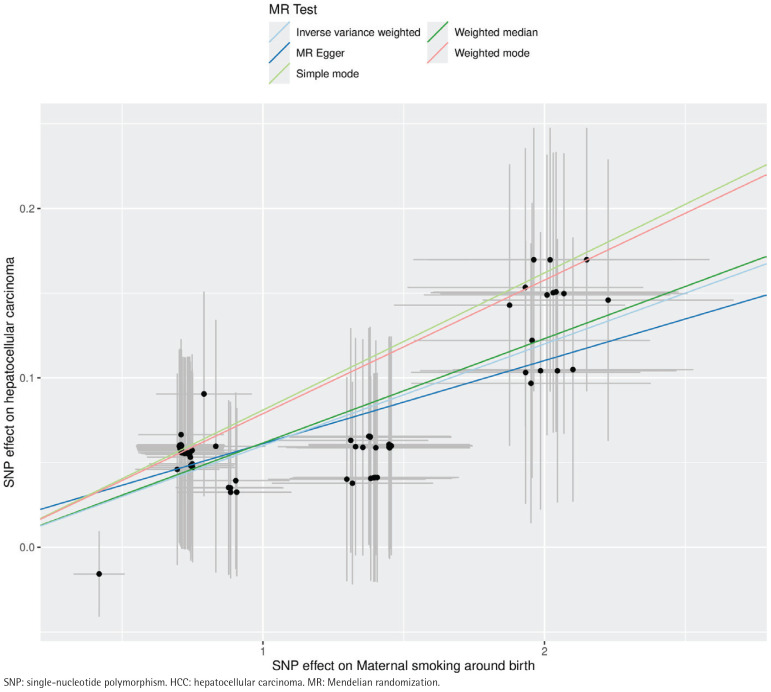
Scatter plot of SNP effect estimates for maternal smoking around birth on the x-axis (in standard deviation units) versus HCC on the y-axis (log odds ratio), including 95% CI. The regression slopes correspond to association estimates from inverse-variance weighted (IVW), weighted median, and MR-Egger analyses, IEU OpenGWAS 2019 and 2020 (N=200017)

MR analyses consistently indicated a positive association between genetically predicted maternal smoking and HCC risk (Table 2). IVW analysis showed a significant association (OR=1.06; 95% CI: 1.05–1.07; p<0.01), supported by the weighted median (OR=1.06; 95% CI: 1.05–1.08) and MR-Egger regression (OR=1.05; 95% CI: 1.02–1.08) (Figure 2). Forest plots (Supplementary file Figure 1) illustrated the consistency of these findings.

**Table 2:** Mendelian randomization results for the association between maternal smoking around birth and HCC, IEU OpenGWAS 2019 and 2020 (N=200017)

*Exposure*	*Outcome*	*Methods*	*β*	*SE*	*OR (95% CI)*	*p*
**Forward MR**						
**Maternal smoking around birth**	Hepatocellular carcinoma	MR Egger	0.05	0.01	1.05 (1.02–1.08)	<0.001
		Weighted median	0.06	0.01	1.06 (1.05–1.08)	<0.001
		IVW	0.06	0.01	1.06 (1.05–1.07)	<0.001
		Simple mode	0.08	0.02	1.08 (1.05–1.12)	<0.001
		Weighted mode	0.08	0.02	1.08 (1.05–1.12)	<0.001
**Reverse MR**						
**Hepatocellular carcinoma**	Maternal smoking around birth	MR Egger	-1.53	0.24	0.22 (0.14-0.35)	<0.001
		Weighted median	0.17	0.04	1.18 (1.08–1.29)	<0.001
		IVW	0.04	0.04	1.04 (0.97–1.12)	0.223
		Simple mode	0.17	0.15	1.19 (0.88–1.59)	0.256
		Weighted mode	0.17	0.15	1.19 (0.89–1.58)	0.242

IVW: inverse variance weighted. SE: standard error.

### Sensitivity analyses

No evidence of directional pleiotropy was observed (MR-Egger intercept p=0.38). Cochran’s Q test confirmed the absence of significant heterogeneity (Table 3). Leave-one-out analyses indicated that no single SNP substantially influenced the overall effect estimates (Supplementary file Figure 2).

**Table 3:** Heterogeneity and pleiotropy tests, IEU OpenGWAS 2019 and 2020 (N=200017)

*Analysis*	*Exposure*	*Outcome*	*Heterogeneity test*				*Pleiotropy test*
			*IVW Q*	*p*	*MR-Egger Q*	*p*	*MR-Egger p*
**Forward MR**	ukb-e-1787_EAS	bbj-a-158	16.21	1	15.44	1	0.38
**Reverse MR**	bbj-a-158	ukb-e-1787_EAS	63.81	1	20.36	1	<0.001

IVW: inverse variance weighted. MR: Mendelian randomization.

### Reverse Mendelian randomization analysis

Reverse MR was performed using 292 independent SNPs associated with HCC at genome-wide significance. IVW results suggested no observed effect of HCC on maternal smoking (Table 2), confirming the directionality of the primary findings. Although heterogeneity was observed in SNP-level leave-one-out analyses, the overall potential effect estimates remained stable.

## DISCUSSION

This bidirectional two-sample MR study provides genetic evidence supporting a potential association between active maternal smoking during pregnancy and an increased risk of HCC in offspring among East Asian populations. Notably, the present investigation specifically focused on active maternal smoking rather than secondhand smoke exposure. A longitudinal cohort study from Japan reported that a considerable proportion of women failed to quit smoking around the time of delivery^24^, indicating that a substantial proportion of pregnant women continue to smoke. Recent evidence has shown that serum levels of nicotine and its major metabolite cotinine are strongly associated with liver cancer risk^8^. Luck et al.^25^ demonstrated that nicotine and cotinine concentrations in maternal blood and amniotic fluid collected between 16 and 24 weeks of gestation were highly associated.

In tobacco smoke, the concentration and biological effects of carbon monoxide (CO) remain of particular concern because CO is present at high levels and is a well-established reproductive toxicant^26^. CO binds tightly to maternal and fetal hemoglobin to form carboxyhemoglobin (COHb), resulting in tissue hypoxia^27^ and stimulating erythropoiesis. Consequently, both pregnant women who smoke and their fetuses typically exhibit elevated hematocrit levels^28^. These alterations in hematocrit and blood viscosity may impair placental perfusion^29^.

Hepatocellular carcinoma (HCC) is characterized by marked hypervascularity and arterialization, with a substantially higher proportion of arterial blood supply than that in normal liver tissue^30^. Given that hypoxia is a potent inducer of tumor angiogenesis, it has been hypothesized that the hypervascularity of HCC is a consequence of hypoxia and that reduced oxygen tension is a key pathogenic feature of HCC^31^. Recent evidence indicates that hepatoblastomas (HBs) and pediatric HCCs together constitute the vast majority of primary malignant liver tumors in children and adolescents/young adults^32^.

### Strengths and limitations

In this study, we conducted a bidirectional, two-sample Mendelian randomization (MR) analysis using large-scale GWAS summary statistics to investigate the potential relationship between maternal smoking around birth and HCC. To date, this represents a comprehensive MR assessment in East Asian populations examining whether genetic variants associated with maternal smoking around birth influence the risk of developing HCC. We rigorously selected 113 SNPs strongly associated with systolic blood pressure as instrumental variables (IVs). These SNPs were chosen under strict criteria, and to further minimize pleiotropic effects, we employed the *FastTraitR* package to exclude variants linked to known confounders, including viral hepatitis. The primary MR analyses were performed using three complementary approaches: inverse-variance weighted (IVW), weighted median, and MR-Egger regression. All three methods consistently indicated a potential positive association between maternal smoking around birth and HCC risk. Sensitivity analyses revealed no evidence of substantial pleiotropy or influential outliers. In contrast, the reverse MR analysis used SNPs associated with HCC as IVs. However, we found no evidence supporting an effect of HCC on maternal smoking around birth, further reinforcing the direction suggested by the forward MR results.

Our MR framework leveraged GWAS summary statistics, enabling strict control for confounding and mitigating reverse causation. The comprehensive datasets provided strong statistical power and broad genomic coverage.

Nevertheless, several limitations should be acknowledged. First, the GWAS data for maternal smoking around birth were derived exclusively from East Asian populations, which may introduce ancestry-related bias and limit the generalizability of our findings to other ethnic groups. Second, in the reverse MR analysis, the MR-Egger model indicated notable horizontal pleiotropy among the IVs; however, such pleiotropy did not materially influence the estimates during maternal–fetal transmission. Third, the two-sample MR design may be vulnerable to over-identification bias, potentially inflating associations between SNPs and the exposure. Fourth, the IEU Open GWAS database does not classify maternal smoking around birth into more granular phenotypic subtypes (e.g. smoking intensity or frequency), preventing subtype-specific evaluations of HCC risk.

## CONCLUSIONS

This bidirectional two-sample MR study provides evidence supporting a potential relationship between maternal smoking around birth and an increased risk of HCC in offspring among East Asians, while accounting for reverse directionality. These findings highlight the complex interplay between the *in utero* environment and long-term offspring health, underscoring the need for strengthened smoking-cessation interventions and further clinical research targeting pregnant women.

## Supplementary Material



## Data Availability

The data supporting this work are provided within the manuscript and the Supplementary file.
