# Supplementary material for: Associations between maternal smoking around birth and hepatocellular carcinoma: A bidirectional two-sample Mendelian randomization study in East Asian populations
Source: Tob Induc Dis. 2026 Apr 28;24:10.18332/tid/218848. doi: 10.18332/tid/218848 (PMC13123645; doi:10.18332/tid/218848)
Supplement: Supplementary file 1 [file TID-24-52-s1.pdf]

Suppl-Table1 Association analysis for maternal smoking around birth -increasing GWAS risk alleles with the HCC, IEU OpenGWAS 2019 and 2020 (N=200,017).

Abbreviations: CHR, chromosome; EA, effect allele; EAF, effect allele frequency; SE, standard

CHR	Position	SNPs	EA	EAF	Maternal smoking around birth			Hepatocellular carcinoma		
					β	SE	P	β	SE	P
2	185299199	rs111615469	C	0.01	1.96	0.43	0.00	0.17	0.08	0.03
2	185301163	rs113620567	C	0.01	2.02	0.43	0.00	0.17	0.08	0.03
2	185310065	rs114447460	G	0.01	2.04	0.44	0.00	0.15	0.08	0.07
2	185314442	rs117252764	C	0.01	2.07	0.44	0.00	0.15	0.08	0.07
2	185299194	rs117623344	T	0.01	2.15	0.44	0.00	0.17	0.08	0.03
2	185397839	rs140668782	C	0.02	1.88	0.41	0.00	0.14	0.08	0.09
2	185406044	rs142879312	G	0.01	1.99	0.43	0.00	0.10	0.08	0.20
2	185321631	rs142980190	G	0.01	2.23	0.45	0.00	0.15	0.08	0.08
2	185431174	rs143426972	G	0.02	1.95	0.43	0.00	0.10	0.08	0.24
2	185312226	rs145165876	T	0.01	2.03	0.44	0.00	0.15	0.08	0.07
2	185392607	rs6712236	T	0.02	2.10	0.43	0.00	0.10	0.08	0.18
2	185390114	rs6724979	A	0.02	2.05	0.42	0.00	0.10	0.08	0.18
2	185304594	rs73039240	C	0.01	1.93	0.42	0.00	0.15	0.08	0.06
2	185372407	rs78754921	A	0.02	1.96	0.42	0.00	0.12	0.08	0.13
2	185317458	rs79009285	T	0.01	2.01	0.44	0.00	0.15	0.08	0.07
2	185386490	rs79121302	G	0.02	1.93	0.41	0.00	0.10	0.08	0.18
6	145762485	rs111294826	T	0.03	1.41	0.29	0.00	0.04	0.06	0.51
6	145741066	rs115817315	C	0.04	1.45	0.29	0.00	0.06	0.07	0.37
6	145774268	rs12524615	G	0.03	1.32	0.29	0.00	0.04	0.06	0.53
6	145770009	rs140330376	A	0.04	1.30	0.28	0.00	0.04	0.06	0.51
6	145752337	rs1949031	T	0.04	1.38	0.29	0.00	0.07	0.06	0.31
6	145743020	rs200417035	T	0.04	1.31	0.28	0.00	0.06	0.07	0.34
6	145750668	rs201615515	T	0.04	1.33	0.28	0.00	0.06	0.06	0.36
6	145751742	rs202227834	G	0.04	1.36	0.27	0.00	0.06	0.06	0.36
6	31860156	rs611572	T	0.07	0.79	0.17	0.00	0.09	0.06	0.13
6	31859947	rs612496	G	0.07	0.79	0.17	0.00	0.09	0.06	0.13
6	145752957	rs6570682	A	0.04	1.40	0.28	0.00	0.06	0.06	0.35
6	145763640	rs74337726	G	0.03	1.39	0.29	0.00	0.04	0.06	0.50
6	145758543	rs74376892	G	0.04	1.45	0.29	0.00	0.06	0.06	0.33
6	145748788	rs74999465	C	0.04	1.46	0.29	0.00	0.06	0.06	0.35
6	145775835	rs75342303	A	0.03	1.38	0.29	0.00	0.04	0.06	0.50
6	145749326	rs75981955	G	0.04	1.38	0.29	0.00	0.06	0.07	0.32
6	145745425	rs76529053	T	0.04	1.45	0.29	0.00	0.06	0.07	0.37
6	145760937	rs76857104	C	0.04	1.45	0.29	0.00	0.06	0.06	0.32
6	145753842	rs77567184	A	0.04	1.38	0.29	0.00	0.07	0.06	0.31
6	145763603	rs77778747	G	0.03	1.40	0.29	0.00	0.04	0.06	0.50
6	145758017	rs77986757	T	0.04	1.45	0.29	0.00	0.06	0.06	0.33
6	145754607	rs78288049	C	0.04	1.45	0.29	0.00	0.06	0.06	0.34
6	145761047	rs79973061	A	0.04	1.45	0.29	0.00	0.06	0.06	0.32
9	124250712	rs10117393	G	0.11	0.71	0.15	0.00	0.06	0.06	0.29
9	124248509	rs10118890	C	0.11	0.71	0.15	0.00	0.06	0.06	0.30
9	124246614	rs10120033	G	0.11	0.71	0.15	0.00	0.06	0.06	0.30
9	124224316	rs10120537	T	0.12	0.71	0.15	0.00	0.07	0.06	0.24
9	124259632	rs10120621	T	0.12	0.70	0.15	0.00	0.06	0.06	0.29

9	124250763	rs10120689	C	0.11	0.71	0.15	0.00	0.06	0.06	0.29
9	124267613	rs10122980	T	0.11	0.71	0.15	0.00	0.06	0.06	0.30
9	124257740	rs10124999	G	0.11	0.71	0.15	0.00	0.06	0.06	0.29
9	124230471	rs10125717	T	0.13	0.70	0.15	0.00	0.05	0.06	0.42
9	124223702	rs1023731	A	0.11	0.75	0.15	0.00	0.06	0.06	0.31
9	124266491	rs10283525	G	0.11	0.71	0.15	0.00	0.06	0.06	0.30
9	124229814	rs10448255	A	0.11	0.74	0.15	0.00	0.06	0.06	0.33
9	124229981	rs10818542	G	0.11	0.74	0.15	0.00	0.06	0.06	0.33
9	124237758	rs10818546	G	0.11	0.74	0.15	0.00	0.05	0.06	0.33
9	124238060	rs10818547	T	0.11	0.73	0.15	0.00	0.06	0.06	0.33
9	124238924	rs10818548	G	0.11	0.73	0.15	0.00	0.06	0.06	0.33
9	124238966	rs10818549	G	0.11	0.73	0.15	0.00	0.06	0.06	0.33
9	124239364	rs10818550	G	0.11	0.72	0.15	0.00	0.06	0.06	0.33
9	124239822	rs10818551	C	0.11	0.72	0.15	0.00	0.06	0.06	0.33
9	124240031	rs10818552	T	0.11	0.71	0.15	0.00	0.06	0.06	0.33
9	124240065	rs10818553	T	0.11	0.71	0.15	0.00	0.06	0.06	0.32
9	124243514	rs10818554	G	0.11	0.71	0.15	0.00	0.06	0.06	0.31
9	124251771	rs10818555	T	0.11	0.71	0.15	0.00	0.06	0.06	0.29
9	124266985	rs10818559	A	0.11	0.71	0.15	0.00	0.06	0.06	0.30
9	124206378	rs10985244	T	0.11	0.74	0.15	0.00	0.05	0.06	0.35
9	124234255	rs10985262	T	0.11	0.74	0.15	0.00	0.06	0.06	0.33
9	124238886	rs10985264	T	0.11	0.73	0.15	0.00	0.06	0.06	0.33
9	124240479	rs10985267	G	0.11	0.72	0.15	0.00	0.06	0.06	0.32
9	124252503	rs10985272	A	0.11	0.71	0.15	0.00	0.06	0.06	0.29
9	124258742	rs10985273	T	0.11	0.71	0.15	0.00	0.06	0.06	0.29
9	124264520	rs10985277	T	0.11	0.71	0.15	0.00	0.06	0.06	0.30
9	124267229	rs111949555	A	0.11	0.71	0.15	0.00	0.06	0.06	0.30
9	124231825	rs12002998	G	0.11	0.74	0.15	0.00	0.06	0.06	0.33
9	108644599	rs12335482	G	0.93	-0.88	0.19	0.00	-0.04	0.05	0.49
9	124250140	rs12339600	T	0.11	0.71	0.15	0.00	0.06	0.06	0.29
9	124256686	rs12341605	T	0.11	0.71	0.15	0.00	0.06	0.06	0.29
9	124256975	rs12341798	T	0.11	0.71	0.15	0.00	0.06	0.06	0.29
9	124257296	rs140818838	A	0.11	0.71	0.15	0.00	0.06	0.06	0.30
9	124255935	rs140867677	A	0.11	0.71	0.15	0.00	0.06	0.06	0.29
9	124203159	rs150737219	A	0.11	0.75	0.15	0.00	0.05	0.06	0.38
9	124255900	rs17417602	C	0.11	0.71	0.15	0.00	0.06	0.06	0.29
9	124268039	rs17417696	C	0.11	0.71	0.15	0.00	0.06	0.06	0.30
9	124200417	rs177698	C	0.11	0.75	0.15	0.00	0.05	0.06	0.40
9	124228191	rs2077776	A	0.11	0.74	0.15	0.00	0.06	0.06	0.33
9	124245435	rs2105288	G	0.11	0.71	0.15	0.00	0.06	0.06	0.30
9	108643176	rs2417654	T	0.92	-0.88	0.19	0.00	-0.04	0.05	0.49
9	124254983	rs28478469	T	0.11	0.70	0.15	0.00	0.06	0.06	0.29
9	124252143	rs28513370	T	0.11	0.71	0.15	0.00	0.06	0.06	0.29
9	124254820	rs28581813	C	0.11	0.71	0.15	0.00	0.06	0.06	0.29
9	108641808	rs3010959	T	0.07	0.90	0.19	0.00	0.04	0.05	0.45
9	124244046	rs4376560	T	0.11	0.71	0.15	0.00	0.06	0.06	0.31
9	124231036	rs4475571	G	0.11	0.74	0.15	0.00	0.06	0.06	0.33
9	124230543	rs4503171	A	0.11	0.74	0.15	0.00	0.06	0.06	0.33
9	108642925	rs4612407	T	0.92	-0.88	0.19	0.00	-0.04	0.05	0.49

9	108645138	rs4742968	G	0.93	-0.89	0.19	0.00	-0.03	0.05	0.52
9	108650257	rs538665565	T	0.93	-0.91	0.19	0.00	-0.03	0.05	0.51
9	108650255	rs546682993	C	0.93	-0.91	0.19	0.00	-0.03	0.05	0.51
9	124235683	rs55955906	T	0.11	0.74	0.15	0.00	0.06	0.06	0.33
9	124235635	rs56127597	A	0.11	0.73	0.15	0.00	0.06	0.06	0.33
9	108650256	rs571348078	C	0.93	-0.91	0.19	0.00	-0.03	0.05	0.51
9	124258201	rs57325794	G	0.11	0.71	0.15	0.00	0.06	0.06	0.29
9	124241214	rs5900488	AC	0.11	0.71	0.15	0.00	0.06	0.06	0.32
9	124242222	rs61499303	C	0.11	0.70	0.15	0.00	0.06	0.06	0.30
9	124227273	rs67848346	A	0.09	0.83	0.18	0.00	0.06	0.07	0.42
9	124201704	rs7044653	T	0.11	0.74	0.15	0.00	0.05	0.06	0.39
9	124232141	rs7045245	C	0.11	0.74	0.15	0.00	0.06	0.06	0.33
9	124261672	rs72762164	C	0.11	0.71	0.15	0.00	0.06	0.06	0.30
9	124269203	rs7849778	G	0.11	0.71	0.15	0.00	0.06	0.06	0.30
9	124265708	rs7858736	T	0.11	0.71	0.15	0.00	0.06	0.06	0.30
9	124235928	rs7859317	T	0.11	0.74	0.15	0.00	0.06	0.06	0.33
9	124241369	rs871670	G	0.11	0.71	0.15	0.00	0.06	0.06	0.31
9	124241314	rs871671	G	0.11	0.71	0.15	0.00	0.06	0.06	0.31
9	124227602	rs9299275	A	0.11	0.73	0.15	0.00	0.06	0.06	0.33
X	103940265	rs55633893	G	0.51	-0.42	0.09	0.00	0.02	0.03	0.53

Abbreviations: CHR, chromosome; EA, effect allele; EAF, effect allele frequency; SE, standard error; SNPs, single-nucleotide polymorphisms; HCC, hepatocellular carcinoma; GWAS, genome-wide association study.

STROBE-MR checklist of recommended items to address in reports of Mendelian randomization studies[1 2]

Item No.	Section		Checklist item	Page No.	Relevant text from manuscript
1	**TITLE and ABSTRACT**		Indicate Mendelian randomization (MR) as the study's design in the title and/or the abstract if that is a main purpose of the study	1	TITLE and ABSTRACT
	**INTRODUCTION**				
2	**Background**		Explain the scientific background and rationale for the reported study. What is the exposure? Is a potential causal relationship between exposure and outcome plausible? Justify why MR is a helpful method to address the study question	2	Line 43-59
3	**Objectives**		State specific objectives clearly, including pre-specified causal hypotheses (if any). State that MR is a method that, under specific assumptions, intends to estimate causal effects	3	Line 72-74
	**METHODS**				
4	**Study design and data sources**		Present key elements of the study design early in the article. Consider including a table listing sources of data for all phases of the study. For each data source contributing to the analysis, describe the following:		
		a)	Setting: Describe the study design and the underlying population, if possible. Describe the setting, locations, and relevant dates, including periods of recruitment, exposure, follow-up, and data collection, when available.	3/5	Line77/Line122-129
		b)	Participants: Give the eligibility criteria, and the sources and methods of selection of participants. Report the sample size, and whether any power or sample size calculations were carried out prior to the main analysis	3	Line81
		c)	Describe measurement, quality control and selection of genetic variants	3/4	Line83-92
		d)	For each exposure, outcome, and other relevant variables, describe methods of assessment and diagnostic criteria for diseases	5	Table1
		e)	Provide details of ethics committee approval and participant informed consent, if relevant		N/A
5	**Assumptions**		Explicitly state the three core IV assumptions for the main analysis (relevance, independence and exclusion restriction) as well assumptions for any additional or sensitivity analysis	4	Line 103
6	**Statistical methods: main**		Describe statistical methods and statistics used		

	**analysis**				
		a)	Describe how quantitative variables were handled in the analyses (i.e., scale, units, model)	4	Line 92-94
		b)	Describe how genetic variants were handled in the analyses and, if applicable, how their weights were selected	4	Line 94-102
		c)	Describe the MR estimator (e.g. two-stage least squares, Wald ratio) and related statistics. Detail the included covariates and, in case of two-sample MR, whether the same covariate set was used for adjustment in the two samples	4	Line 104-105
		d)	Explain how missing data were addressed	3/4	Line 90-92
		e)	If applicable, indicate how multiple testing was addressed	4	Line110
7	**Assessment of assumptions**		Describe any methods or prior knowledge used to assess the assumptions or justify their validity	4	Line 104-109
8	**Sensitivity analyses and additional analyses**		Describe any sensitivity analyses or additional analyses performed (e.g. comparison of effect estimates from different approaches, independent replication, bias analytic techniques, validation of instruments, simulations)	4	Line 116-118
9	**Software and pre-registration**				
		a)	Name statistical software and package(s), including version and settings used	4	Line 104-105
		b)	State whether the study protocol and details were pre-registered (as well as when and where)		N/A
	**RESULTS**				
10	**Descriptive data**				
		a)	Report the numbers of individuals at each stage of included studies and reasons for exclusion. Consider use of a flow diagram	5	Line 136-138, Suppl-Table 1
		b)	Report summary statistics for phenotypic exposure(s), outcome(s), and other relevant variables (e.g. means, SDs, proportions)	5	Line 144-150, Table2
		c)	If the data sources include meta-analyses of previous studies, provide the assessments of heterogeneity across these studies		N/A
		d)	For two-sample MR:<br>i. Provide justification of the similarity of the genetic variant-exposure associations	5	Line 136-138, Suppl-Table 1

			between the exposure and outcome samples<br>ii. Provide information on the number of individuals who overlap between the exposure and outcome studies		
11	**Main results**				
		a)	Report the associations between genetic variant and exposure, and between genetic variant and outcome, preferably on an interpretable scale	5	Line 144-150, Table2
		b)	Report MR estimates of the relationship between exposure and outcome, and the measures of uncertainty from the MR analysis, on an interpretable scale, such as odds ratio or relative risk per SD difference	5	Table 2
		c)	If relevant, consider translating estimates of relative risk into absolute risk for a meaningful time period		N/A
		d)	Consider plots to visualize results (e.g. forest plot, scatterplot of associations between genetic variants and outcome versus between genetic variants and exposure)		N/A
12	**Assessment of assumptions**				
		a)	Report the assessment of the validity of the assumptions	6	Line 156-159
		b)	Report any additional statistics (e.g., assessments of heterogeneity across genetic variants, such as I^2, Q statistic or E-value)	6	Table 3
13	**Sensitivity analyses and additional analyses**				
		a)	Report any sensitivity analyses to assess the robustness of the main results to violations of the assumptions	6	Line 156-159
		b)	Report results from other sensitivity analyses or additional analyses	6	Line 156-159
		c)	Report any assessment of direction of causal relationship (e.g., bidirectional MR)	6	Table 3
		d)	When relevant, report and compare with estimates from non-MR analyses		N/A
		e)	Consider additional plots to visualize results (e.g., leave-one-out analyses)		N/A
	**DISCUSSION**				
14	**Key results**		Summarize key results with reference to study objectives	6	Line 173-176
15	**Limitations**		Discuss limitations of the study, taking into account the validity of the IV assumptions,	8	Line 223-238

			other sources of potential bias, and imprecision. Discuss both direction and magnitude of any potential bias and any efforts to address them		
16	**Interpretation**				
		a)	Meaning: Give a cautious overall interpretation of results in the context of their limitations and in comparison with other studies	7	Line 204-207
		b)	Mechanism: Discuss underlying biological mechanisms that could drive a potential causal relationship between the investigated exposure and the outcome, and whether the gene-environment equivalence assumption is reasonable. Use causal language carefully, clarifying that IV estimates may provide causal effects only under certain assumptions	7	Line 186-192
		c)	Clinical relevance: Discuss whether the results have clinical or public policy relevance, and to what extent they inform effect sizes of possible interventions	9	Line 244-247
17	**Generalizability**		Discuss the generalizability of the study results (a) to other populations, (b) across other exposure periods/timings, and (c) across other levels of exposure	8	Line 227-230
	**OTHER INFORMATION**				
18	**Funding**		Describe sources of funding and the role of funders in the present study and, if applicable, sources of funding for the databases and original study or studies on which the present study is based	15	Funding
19	**Data and data sharing**		Provide the data used to perform all analyses or report where and how the data can be accessed, and reference these sources in the article. Provide the statistical code needed to reproduce the results in the article, or report whether the code is publicly accessible and if so, where	15	Data Availability Statement
20	**Conflicts of Interest**		All authors should declare all potential conflicts of interest	15	Disclosure statement

This checklist is copyrighted by the Equator Network under the Creative Commons Attribution 3.0 Unported (CC BY 3.0) license.

1. Skrivankova VW, Richmond RC, Woolf BAR, Yarmolinsky J, Davies NM, Swanson SA, et al. Strengthening the Reporting of Observational Studies in Epidemiology using Mendelian Randomization (STROBE-MR) Statement. JAMA. 2021;under review.

2. Skrivankova VW, Richmond RC, Woolf BAR, Davies NM, Swanson SA, VanderWeele TJ, et al. Strengthening the Reporting of Observational Studies in Epidemiology using Mendelian Randomisation (STROBE-MR): Explanation and Elaboration. BMJ. 2021;375:n2233.

Suppl-file Figure 1 Forest plot of SNP-specific associations between maternal smoking around birth and HCC risk. Black points represent the OR for offspring HCC per SD increase in maternal smoking around birth, data from 2019 and 2020 (N=2,406 for Maternal smoking around birth; N= 197,611 for Hepatocellular carcinoma). (SNP, single-nucleotide polymorphism; HCC, hepatocellular carcinoma; OR, odds ratio; SD, standard deviation.)

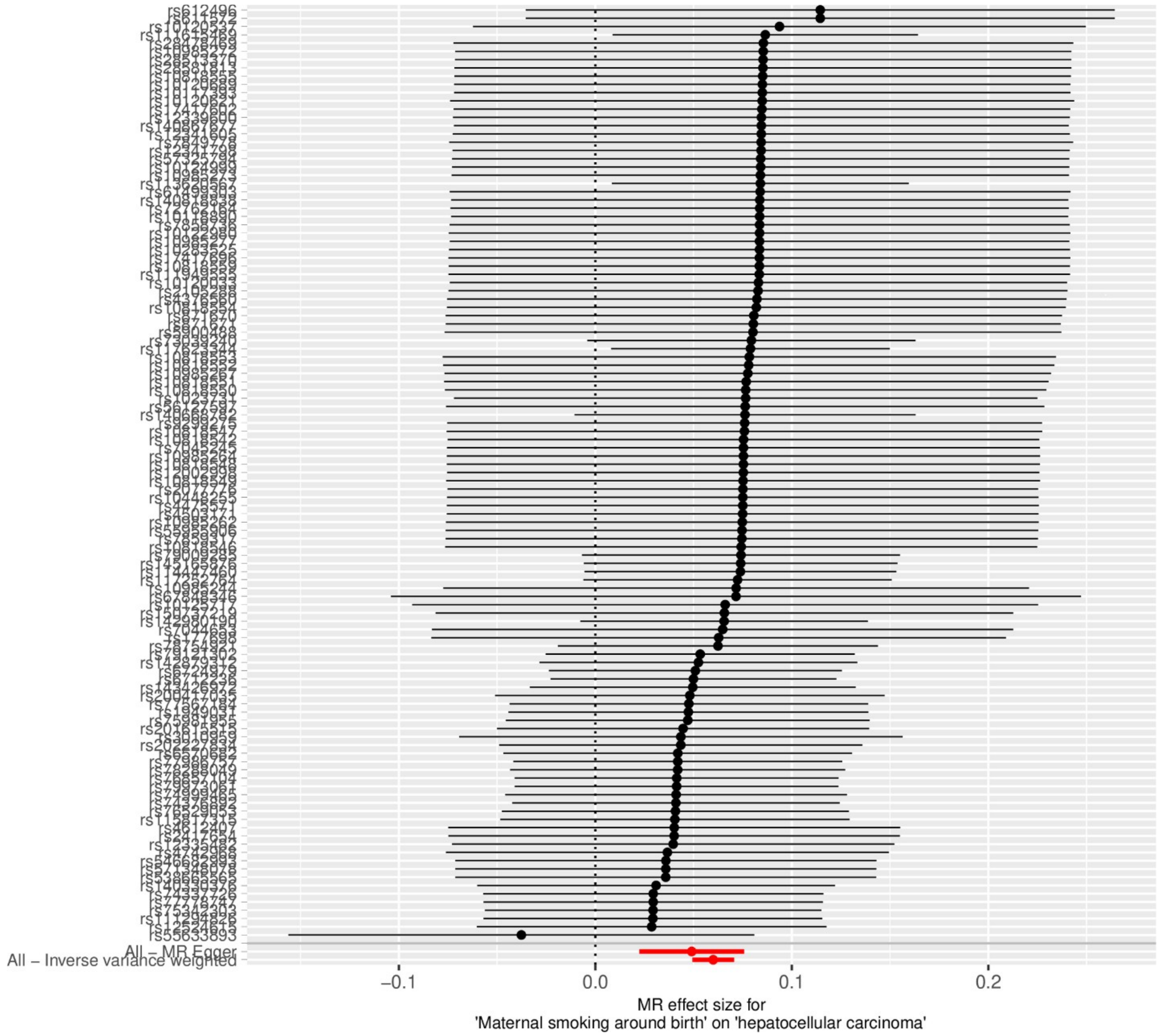

Suppl-file Figure 2 Leave-one-out analysis of SNPs associated with maternal smoking around birth and HCC risk. Each black point represents the result of the IVW MR method applied to estimate the effect of Maternal smoking around birth on HCC, excluding a particular SNP, IEU OpenGWAS 2019 and 2020 (N=200,017). (SNPs, single-nucleotide polymorphisms; HCC, hepatocellular carcinoma; MR, mendelian randomization; IVW, inverse-variance weighted.)

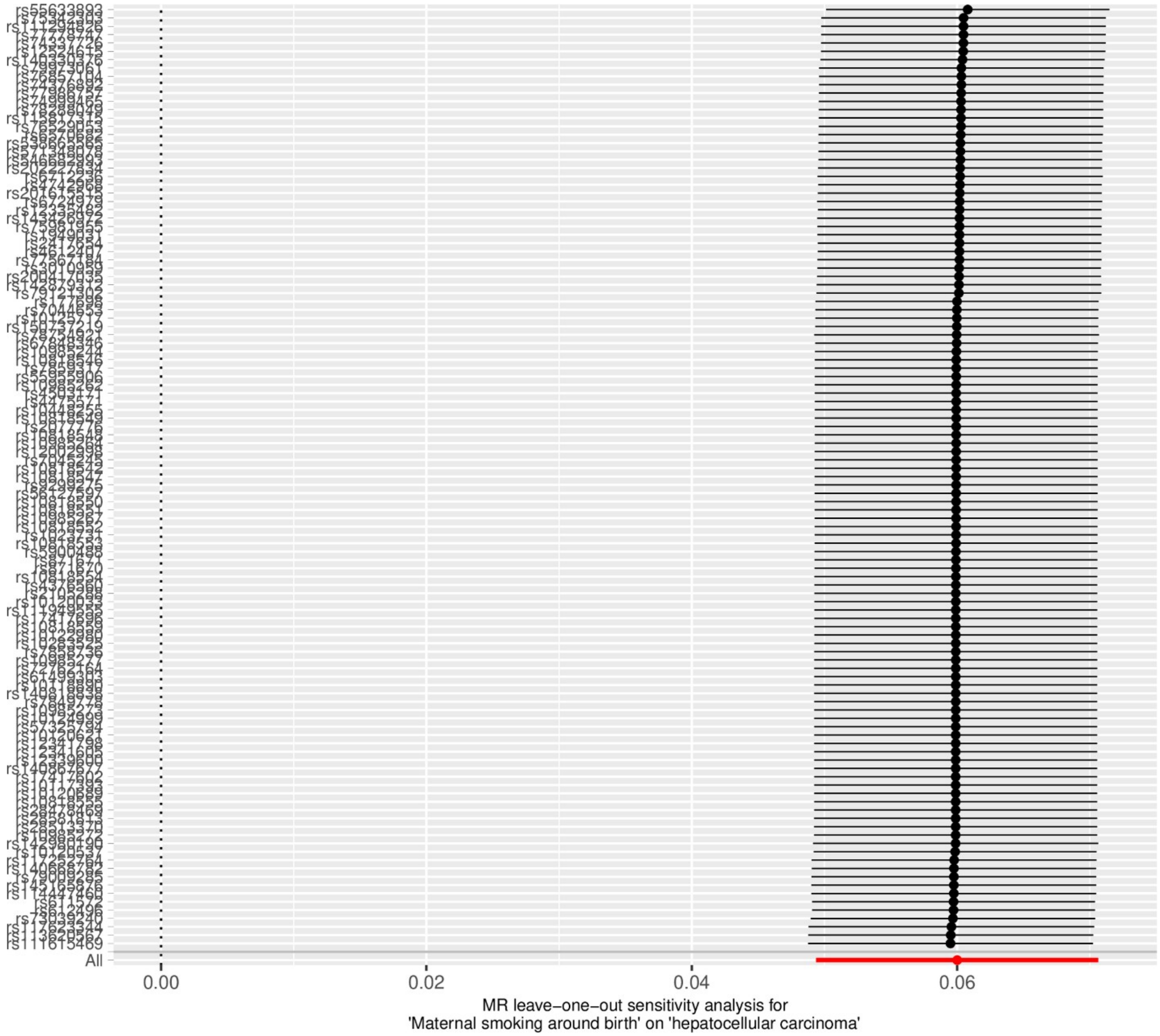

© 2026 Liu W. et al.
